# Aging alters Hv1‐mediated microglial polarization and enhances neuroinflammation after peripheral surgery

**DOI:** 10.1111/cns.13271

**Published:** 2019-11-27

**Authors:** Zhi‐jing Zhang, Xin‐xun Zheng, Xin‐yun Zhang, Yi Zhang, Bao‐yi Huang, Tao Luo

**Affiliations:** ^1^ Department of Anesthesiology Peking University Shenzhen Hospital Shenzhen China; ^2^ Shantou University Medical College Shantou China; ^3^ Anhui Medical University Hefei China

**Keywords:** aging, Hv1, microglial polarization, neuroinflammation

## Abstract

Perioperative neurocognitive disorders have been widely recognized as common adverse events after surgical intervention. Aging is one of the most important independent risk factors for worsened cognitive outcome, and this deterioration is linked to exacerbated microglia‐mediated neuroinflammation in the aged brain. Under pathological stimulation, microglia are capable of polarizing toward proinflammatory M1 and anti‐inflammatory M2 phenotypes. In the present study, we examined how aging affects microglial responses and neuroinflammation following peripheral surgery. Adult (2‐3 months) and aged (18 months old) male C57/BL6 mice were subjected to tibial fracture or sham surgery. Aged mice exhibited higher level of tumor necrosis factor‐α (TNF‐α) and interleukin‐1β (IL‐1β) in the hippocampus. The expression of synaptic protein synaptophysin (SYP) was also markedly reduced in the aged brain after the surgery. Both adult and aged mice showed significant increases in M1 microglial polarization (CD16/32). In contrast, tibial fracture surgery induced a decreased M2 microglial polarization (CD206, Ym1/2, Arg1) in aged brain but enhanced M2 microglial polarization in adult brain. Aged mice have upregulated voltage‐gated proton channel (Hv1) and nicotinamide adenine dinucleotide phosphate (NADPH) oxidase subunit expression compared with adult mice. The percentage of CD16/32‐positive M1 microglia colabeling with Hv1 was higher in aged mice after tibial fracture surgery. Thus, Hv1/NADPH oxidase upregulation in the aged brain may shift the dynamic equilibrium of microglial activation toward M1 polarization and exaggerate postoperative neuroinflammatory responses after peripheral surgical intervention.

**Abbreviations**
Arg1: Arginase 1
CNS: central nervous system
DAPI: 4′,6‐diamidino‐2‐phenylindole
dMCAO: distal middle cerebral artery occlusion
Iba1: ionized calcium‐binding adapter molecule‐1
IL‐1β: interleukin‐1β
iNOS: inducible nitric oxide synthase
MBP: myelin basic protein
NADPH: nicotinamide adenine dinucleotide phosphate
PBS: phosphate‐buffered saline
PFA: paraformaldehyde
POCD: postoperative cognitive dysfunction
POD: postoperative delirium
PVDF: polyvinylidene fluoride
RIPA: radioimmunoprecipitation assay
SYP: synaptophysin
TBI: traumatic brain injury
TNF‐α: tumor necrosis factor‐α

## 1 INTRODUCTION

It is estimated that 234.2 million major surgical procedures are performed every year worldwide.^1^ Postoperative delirium (POD) and/or postoperative cognitive dysfunction (POCD) have been widely recognized as common adverse events after surgical intervention, with reported POD incidence ranges from 13.2% to 41.7% and POCD incidence ranges from 8.9% to 46.1%.^2^ The prevalence of POD and POCD is associated with higher morbidity, mortality, and greater utilization of social financial assistance.^2^ Aging is one of the most important independent risk factors for both POD and POCD.^3^, ^4^ In animal models of peripheral surgical intervention (tibial fracture, open abdominal surgery, etc), aged animals exhibit worsened cognitive outcome, and this deterioration is linked to exacerbated neuroinflammation and macrophage/microglial activation in the aged brain.^5^, ^6^, ^7^, ^8^ Despite these observations, little is known about the mechanisms involved in age‐related pathology following peripheral surgical intervention.

Microglia/macrophage play a major role in host defense and tissue repair in the central nervous system (CNS). Under physiological conditions, microglia are typically found in a resting state. However, in response to injury, infection, or inflammation, microglia rapidly transform into an activated state. Classically activated M1 microglia/macrophages are capable of producing various proinflammatory cytokines, including IL‐1β, IL‐6, TNF‐α, and expressing cell‐surface marker CD16/32,^9^, ^10^ whereas alternatively activated M2 microglia/macrophages are anti‐inflammatory and protective with expressing YM1/2, CD206, Arginase (Arg)1, etc.^9^ Emerging evidence now supports M1/M2 microglia/macrophage polarization alters in several types of acute CNS injuries, including traumatic brain injury, spinal cord injury, and stroke.^11^, ^12^, ^13^, ^14^ Aging is suggested to alter the balance between destructive M1 and protective M2 phenotypes, thus contributed to enhanced neuroinflammation and tissue damage following CNS injuries.^15^, ^16^ However, the effect of age on brain microglial polarization in response to peripheral surgical intervention is unclear.

The voltage‐gated proton channel Hv1 is selectively expressed in microglial cells of the brain.^17^ Hv1 is required for NADPH‐dependent reactive molecules containing oxygen generation.^18^ NADPH‐mediated ischemic brain injury and neuronal damage can be inhibited by suppression of microglia Hv1.^17^ In the present studies, we hypothesize that microglial/macrophage activation in the aged brain after peripheral tibial fracture surgery is associated with an imbalance in the M1 and M2 microglia/macrophage activation phenotypes compared to adult health brain. These changes may be associated with age‐related changes in Hv1 as well as NADPH expression which contribute to acquiring and retaining microglia/macrophage in a M1 activation state, thereby enhancing proinflammatory responses and synaptic dysfunction in the aged brain after peripheral surgical intervention.

## 2 MATERIALS AND METHODS

### 2.1 Animals

Both adult (8‐10 weeks old) and aged (18 months old) male C57/BL6 mice, obtained from Model Animal Research Center of Nanjing University, were housed in a temperature‐controlled room on a 12‐h light and 12‐h dark cycle with free access to food and water. All experimental procedures were performed in accordance with the Institutional Health Guide for Care and Use of Laboratory Animals.

### 2.2 Tibial fracture

Animals received an open tibial fracture of the right hind paw with an intramedullary fixation in aseptic conditions under isoflurane anesthesia. The tibial fracture model was established as previously reported.^19^ In brief, anesthesia consisted of induction with 3.0% isoflurane followed by maintenance with 1.5% isoflurane carried by 100% oxygen. The loss of pedal reflex is used to established surgical anesthesia. Animals were maintained at 37°C body temperature by a warming blanket during surgery. A small incision was performed on the right tibial plateau, followed by the insertion of a 7.0 mm pin into the intramedullary canal. The periosteum was stripped, and osteotomy was created at the junction of the middle and distal thirds of the tibial, under direct vision using rotor. After surgery, the broken skin was sutured, and the animals were maintained in a warmed cage. The same anesthesia and surgery procedures, except tibial fracture, were performed in the sham‐operated group.

### 2.3 Tissue processing and immunofluorescence

Six hours or 72 hours postsurgery, mice in sham or surgery group were sacrificed, respectively, by an overdose i.p. injection of ketamine (120 mg/kg) and xylazine (10 mg/kg) and then transcardial perfusion with precooled saline and fixed with 4% paraformaldehyde (PFA). The whole brain was dissected and fixed overnight in 4% PFA and then dehydrated by immersion in 18% sucrose at 4°C. The brains from the different experimental groups were randomly distributed (by experimenters blinded to group inclusion). Each block was cut (20 µm) and frozen at 4°C before staining.

For immunofluorescence, brain sections were warmed for 1 hour at 37°C and washed in 0.1 mol/L phosphate‐buffered saline (PBS) for 5 minutes, four times. Then, slides were incubated in 5% BSA blocking buffer at room temperature for 1.5 hours, followed by incubated 48 hours at 4°C in 0.5% blocking buffer containing the following primary antibodies: anti‐CD16/32 (1:50, Abcam), anti‐CD206 (1:40, R&D Systems), anti‐Iba1 (1:250, Wako), and anti‐Hv1 (1:50, Sigma‐Aldrich). Next, slides were rinsed in 0.1 mol/L PBS and then incubated with secondary antibodies (Jackson ImmunoResearch Laboratories Inc) at room temperature for 2 hours, followed by mounting with glass coverslips (Citotest, 10212450C).

All fluorescence images were obtained using BX530 Olympus microscopy equipped with an Olympus DP47 digital camera and processed uniformly using Adobe Photoshop CS5. The microglial polarization was analyzed by counting the CD16/32+/Iba1 + and CD206+/Iba1 + cells of three independent microscopic fields in the hippocampal dentate gyrus area of each brain section. The number of positive cells in each section was determined by its average number in the three visual whole fields, and the average number of three sections was taken as the final number of each animal. A researcher blind to all samples’ case histories conducted the data analysis.

### 2.4 Western blot analysis

At 6 hours or 72 hours postsurgery, animals were sacrificed and the hippocampus was rapidly separated and frozen at −80°C. The samples were homogenized in RIPA Lysis Buffer (Beyotime, P0013C, Shanghai, China) containing protease inhibitor cocktail (MCE, HY‐K0010, Shanghai, China). Tissue lysates were centrifuged at 12 000 *g* for 10 minutes at 4°C. The supernatants were collected, and the protein concentrations were determined by using a BCA protein assay kit (Biosharp, BL521A). 30 µg of protein was separated by 10% SDS‐PAGE and then transferred onto polyvinylidene fluoride (PVDF) membranes. After being placed in blocking buffer, the blots were incubated overnight at 4°C with the following primary antibodies: anti‐TNF‐α (1:200, Abcam), anti‐IL‐1β (1:1000, Proteintech), anti‐synaptophysin (1:20 000, Abcam), anti‐Iba1 (1:500, Santa Cruz Biotechnology, Inc), anti‐YM1/2 (1:20 000, Abcam), anti‐Arg1 (1:1000, Abcam), anti‐Hv1 (1:1000, Sigma‐Aldrich), anti‐Gp91phox (1:750, Santa Cruz Biotechnology, Inc), anti‐P22phox (1:750, Santa Cruz Biotechnology, Inc), anti‐P47phox (1:500, Santa Cruz Biotechnology, Inc), anti‐P67phox (1:500, Santa Cruz Biotechnology, Inc), anti‐P40phox (1:500, Santa Cruz Biotechnology, Inc), or anti‐β‐actin (1:4000, Cell Signaling Technology, Inc) and then with horseradish peroxidase–conjugated secondary antibodies (Biolong). ECL Western blotting detection reagents (Tanon 5200) were used for visualization of the protein bands. The density of the protein band was detected by image analysis system (Image‐Pro Plus version 6.0), and the ratio of the interest proteins to β‐actin was calculated.

### 2.5 Statistical analyses

Statistical analyses were performed with the Prism 5 (GraphPad Software). All the data are represented as means ± standard error of the mean (SEM). For multiple comparisons (surgery × age), the two‐way ANOVA was followed by a Bonferroni post hoc test. Remaining data were analyzed with Student's *t* test. A *P* value < 0.05 was considered statistically significant.

## 3 RESULTS

### 3.1 Neuroinflammation is increased with aging after peripheral surgery

**Figure 1:**
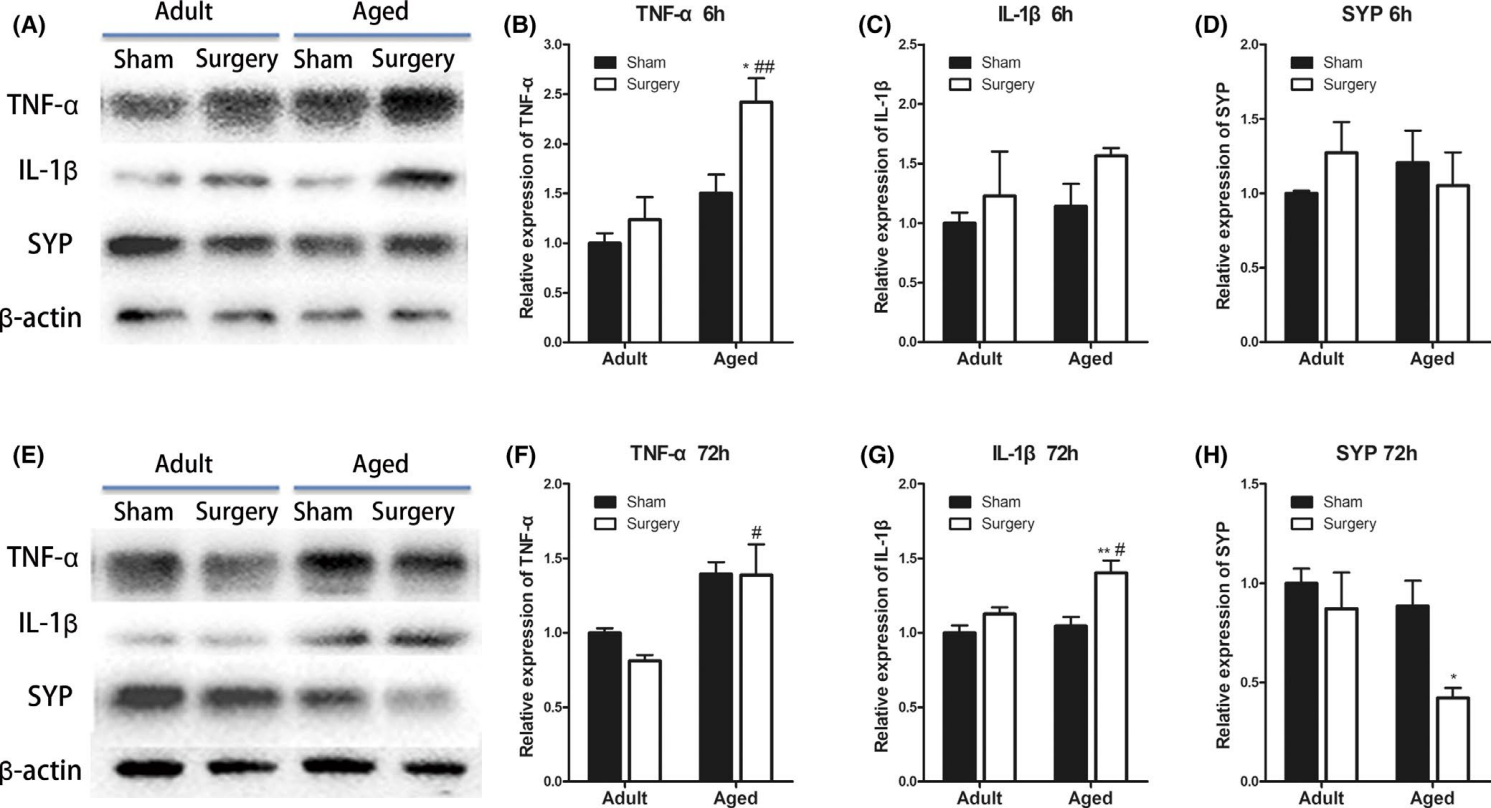
Neuroinflammation is increased with age after peripheral surgery. A‐B, Representative Western blot images of hippocampal TNF‐α, IL‐1β, SYP, and β‐actin expression at 6 and 72 h after tibial fracture. C‐D, The expression of TNF‐α increased in the aged hippocampus at both 6 and 72 h after surgery. E‐F, The level of IL‐1β increased in the aged hippocampus at 72 h after surgery. G‐H, The expression of synaptic protein SYP decreased in the aged hippocampus at 72 h after surgery. All data are presented as the mean ± SEM. n = 4‐7; *P < .05, **P < .01 vs age‐matched sham; #*P* < .05, ##*P* < .01 vs adult surgery, two‐way ANOVA followed by Bonferroni's post hoc test

Peripheral surgery‐induced innate immune response triggers inflammatory process in the hippocampus and subsequent memory impairment in adult animals.^20^ We first determined whether activation of the peripheral innate immune system with tibial fracture surgery would induce a different inflammatory response in the brain of aged mice compared with adults. We found that the peripheral surgery triggered very mild increase in the levels of TNF‐α and IL‐1β in the hippocampus of adult mice as compared to that of sham operation (n = 3‐6; *P* > .05; Figure 1A‐F). In the aged mice, however, tibial fracture significantly upregulated TNF‐α expression both at 6 h (n = 4‐5; *P* < .05 vs aged sham; Figure 1C) and 72 h (n = 4‐7; *P* < .05 vs adult surgery; Figure 1D) after surgery. Moreover, the level of IL‐1β, the downstream cytokine of TNF‐α, also increased in aged brain at 72 hours postsurgery (n = 4‐7; *P* < .01 vs aged sham; *P* < .05 vs adult surgery; Figure 1F). The expression of SYP, a synaptic protein, decreased in aged hippocampus 72 hours after surgery (n = 4‐5; *P* < .05 vs aged sham; Figure 1H). These data suggested that peripheral surgery mainly caused exacerbated neuroinflammation and synaptic function disorder in the aged mice, but not in the adult mice.

### 3.2 Microglial activation is altered with aging following peripheral surgery

**Figure 2:**
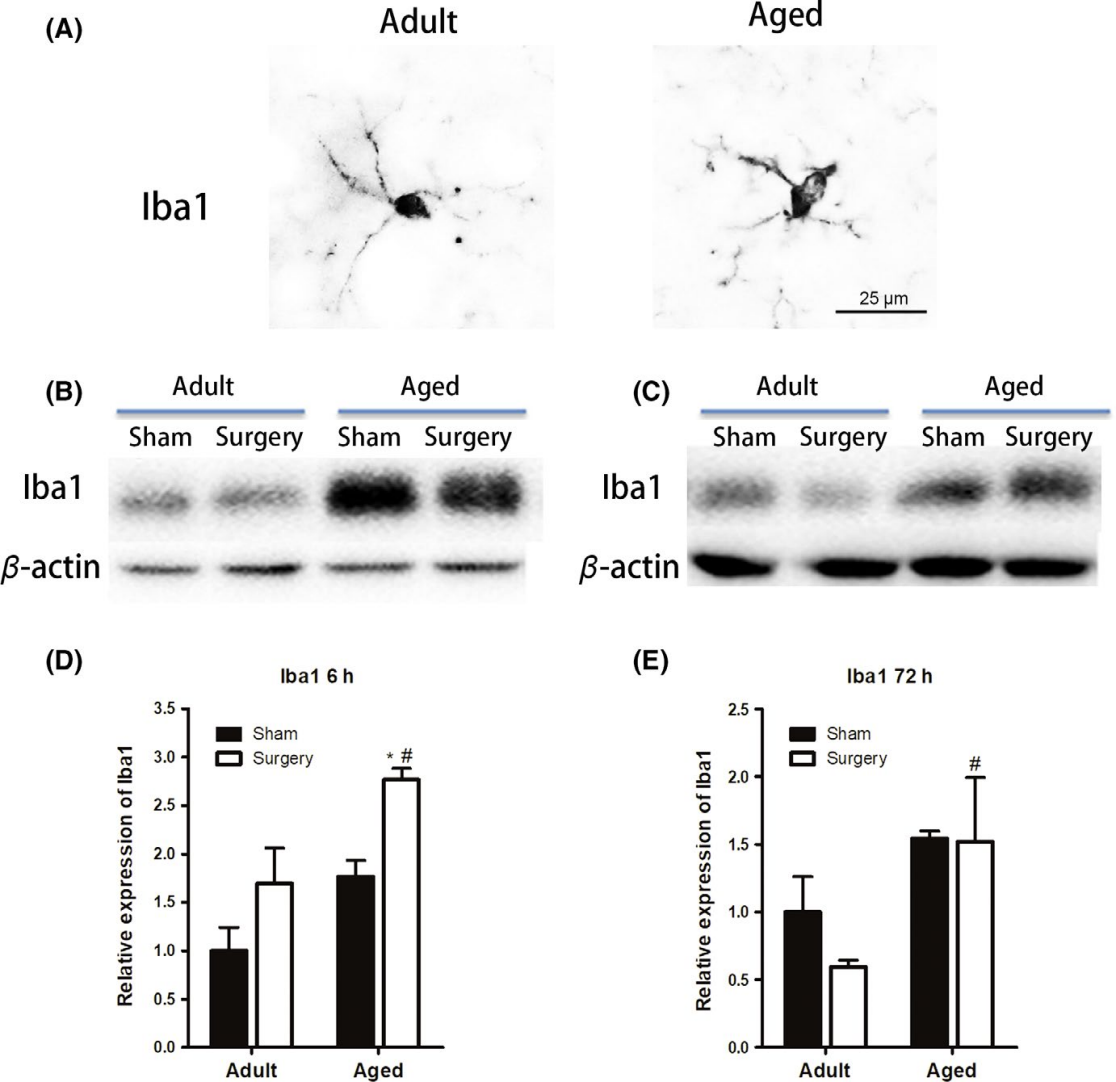
Microglial activation is altered with age following surgery. A, Microglial morphology in adult and aged brain with Iba1 staining (scale bar, 25 μm). Microglial in young adult brain showed small cell body with elongated and thin projections, while multiple short processes that form thick bundles around enlarged cell bodies were presented in the microglia from aged brain. B‐E, Western blot analysis revealed that aged mice had significantly increased Iba1 expression at 6 h after tibial fracture surgery. And the level of Iba1 was consistently higher in the aged than adult mice after tibial fracture surgery. All data are presented as the mean ± SEM. n = 4‐5; *P < .05 vs age‐matched sham; #*P* < .05, vs adult surgery, two‐way ANOVA followed by Bonferroni's post hoc test

Microglia are commonly activated in the early state of the CNS to a wide variety of pathological stimuli, and it may reflect the degree of severity of trauma.^21^ To determine whether aged brain showed differences in microglial activation, hippocampus tissue from adult and aged mice was immunoassayed with the microglial marker ionized calcium‐binding adapter molecule‐1 (Iba1). We found that microglia in young adult brain showed small cell body with elongated and thin projections, while multiple short processes that form thick bundles around enlarged cell bodies were presented in the microglia from aged brain (Figure 2A). Meanwhile, in contrast to adult, aged microglia were more vulnerable to surgery with significantly increased Iba1 expression by 1.5‐fold at 6 hours after peripheral surgical intervention (n = 3‐4; *P* < .05 vs aged sham; Figure 2B,D). And the level of Iba1 was consistently higher in the aged than adult after tibial fracture surgery (n = 3‐4; *P* < .05 vs adult surgery; Figure 2C,E). These data suggested that microglia in the aged brain remained in a pre‐activated condition, and the peripheral surgery further enhanced hippocampus microglial activation.

### 3.3 Aging enhanced M1 microglial activation after peripheral surgery

**Figure 3:**
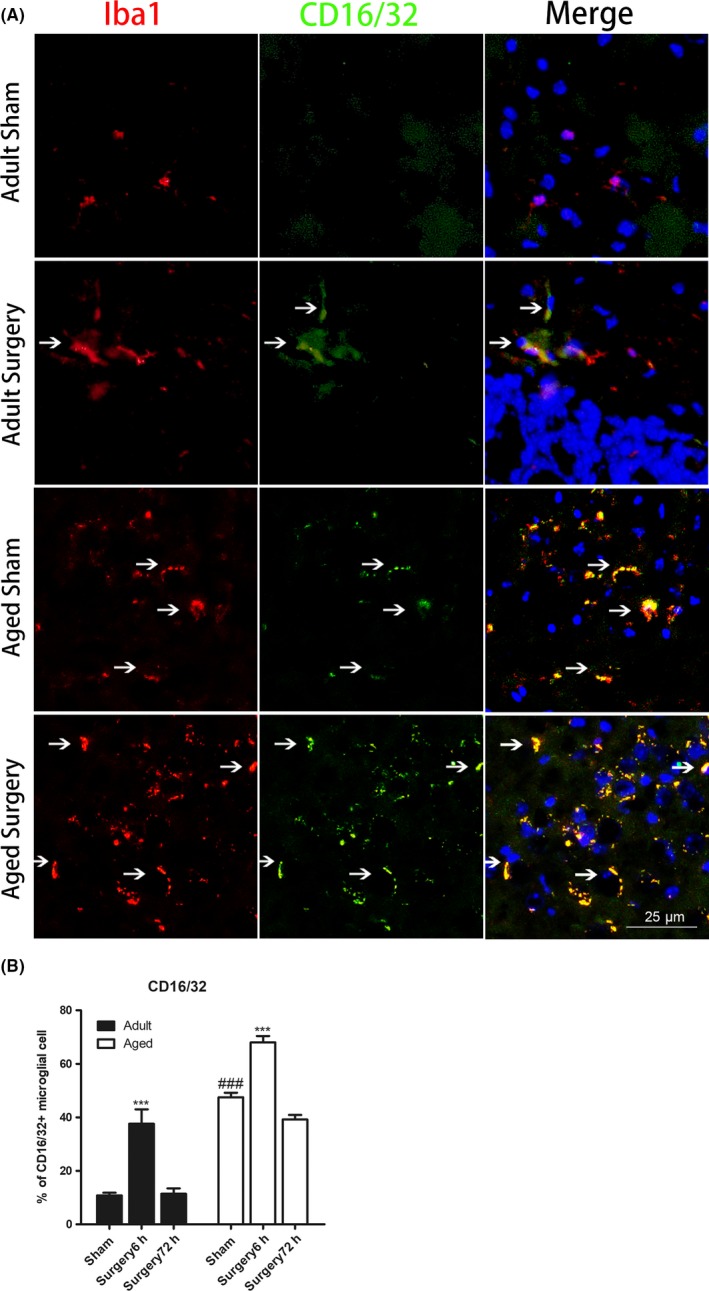
Aging enhanced M1 microglial activation after peripheral surgery. A, Representative immunofluorescent images of the hippocampal dentate gyrus showing that aging enhanced M2 microglial activation at 6 h after peripheral surgery (scale bar, 25 μm). Brain sections were stained with Iba1 (red), M1 phenotype microglial marker CD16/32 (green), and DAPI (blue). B, Immunostaining analysis showing that the percentage of CD16/32‐positive microglia was higher in aged sham as compared with adult sham‐operated mice. Following tibial fracture, the percentage of CD16/32‐positive microglia significantly increased at 6 h and returned to baseline level 72 h after surgery in both adult and aged mice. All data are presented as the mean ± SEM. n = 4‐5; ***P < .001 vs age‐matched sham; ###*P* < .001, vs adult sham, two‐way ANOVA followed by Bonferroni's post hoc test

Microglia may polarize to proinflammatory (M1) or anti‐inflammatory (M2) phenotype in response to pathological stimulation. To further study the phenotype of microglial activation after peripheral trauma, we examined the expression of M1 (CD16/CD32) markers in Iba1 + microglia by double immunofluorescent staining. As can be seen in Figure 3, aged mice demonstrated higher baseline level of CD16/32 + microglia in the hippocampal dentate gyrus area as compared with adult (n = 4‐6; *P* < .001 vs adult sham; Figure 3A,B), indicating that aging itself switches microglial polarization states toward M1 phenotype. Following tibial fracture, the percentage of CD16/32 + microglia significantly increased at 6 hours and returned to baseline level 72 hours after surgery in both adult (n = 5‐6; *P* < .001 vs adult sham at 6 hr; Figure 3B) and aged mice (n = 4; *P* < .001 vs aged sham at 6 hr; Figure 3B).

### 3.4 Aging diminished M2 microglial activation after peripheral surgery

**Figure 4:**
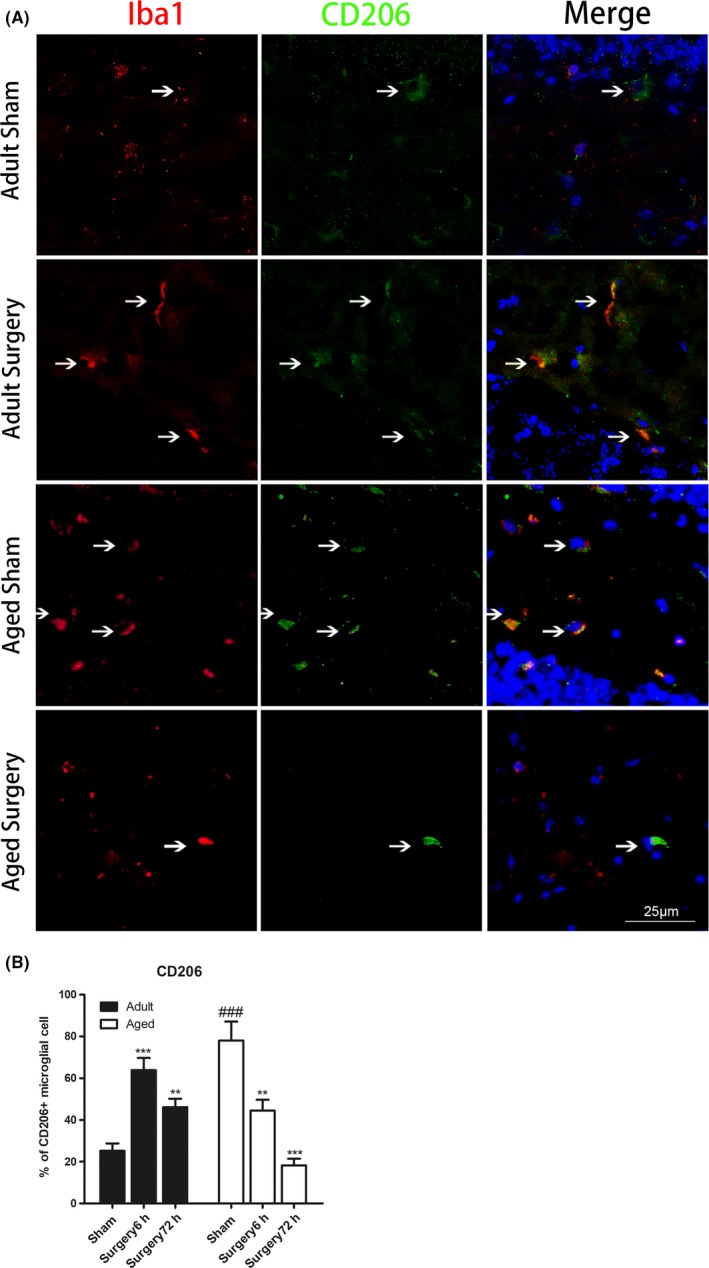
M2 microglial polarization is reduced in aged animals after peripheral surgery. A, Representative immunofluorescent images of the hippocampal dentate gyrus showing that aging reduced M2 microglial activation at 6 h after peripheral surgery (scale bar, 25μm). Brain sections were stained with Iba1 (red), M2 phenotype microglial marker CD206 (green), and DAPI (blue). B, Immunostaining analysis showing that the percentage of CD206‐positive microglia was increased in adult mice at 6 and 72 h following tibial fracture surgery. In contrast, CD206‐positive microglia in aged brain significantly decreased at 6 and 72 h after surgery. All data are presented as the mean ± SEM. n = 3‐8; **P < .01, ***P < .001, vs age‐matched sham; ###*P* < .001, vs adult sham, two‐way ANOVA followed by Bonferroni's post hoc test

**Figure 5:**
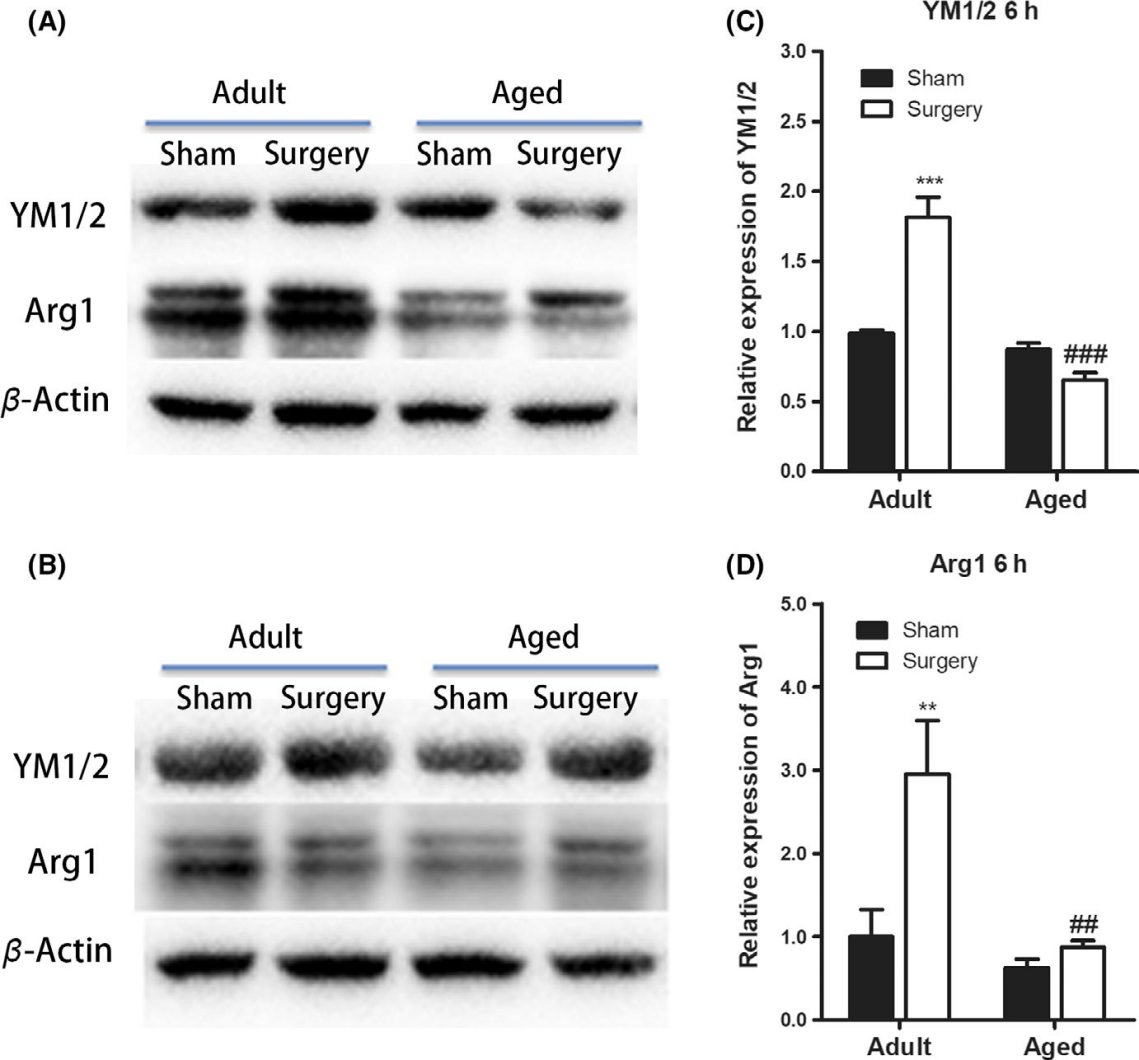
Aging diminished M2 microglial activation after peripheral surgery. A‐B, Representative Western blot images of hippocampal YM1/2, Arg1, and β‐actin expression at 6 and 72 h after tibial fracture. C‐D, The expressions of YM1/2 and Arg1 increased at 6 h after surgery in adult mice while persistently decreased in aged mice. There were no statistical differences at 72 h after surgery in both adult and aged mice (data not shown). All data are presented as the mean ± SEM. n = 3‐6; **P < .01, ***P < .001 vs age‐matched sham; ##*P* < .01, ###*P* < .001, vs adult surgery, two‐way ANOVA followed by Bonferroni's post hoc test

The expression of M2 markers (CD206) in Iba1 + microglia in the hippocampal dentate gyrus area is shown in Figure 4. The CD206 + microglia were increased in adult mice at 6 hours (n = 7; *P* < .001 vs adult sham; Figure 4B) and 72 hours (n = 7‐8; *P* < .01 vs adult sham; Figure 4B) following tibial fracture surgery. In contrast, CD206 + microglia in the aged brain significantly decreased at 6 and 72 hours postsurgery (n = 3‐5; *P* < .01 vs aged sham at 6 hours; *P* < .001 vs aged sham at 72 hours; Figure 4B). Western blot of M2 markers further confirmed that the postsurgical expressions of YM1/2 and Arg1 increased by 1.8‐ and 3‐fold, respectively, over the basal level of adult animals (YM1/2: n = 3‐6; *P* < .001 vs adult sham; Arg1: n = 3‐6; *P* < .01 vs adult sham; Figure 5). Meanwhile, in the aged brain, there was less YM1/2 and Arg1 expression after surgery (YM1/2: n = 4‐6; *P* < .001 vs adult surgery; Arg1: n = 4‐6; *P* < .01 vs adult surgery; Figure 5).

### 3.5 Hv1 and NADPH component activation is altered with aging

**Figure 6:**
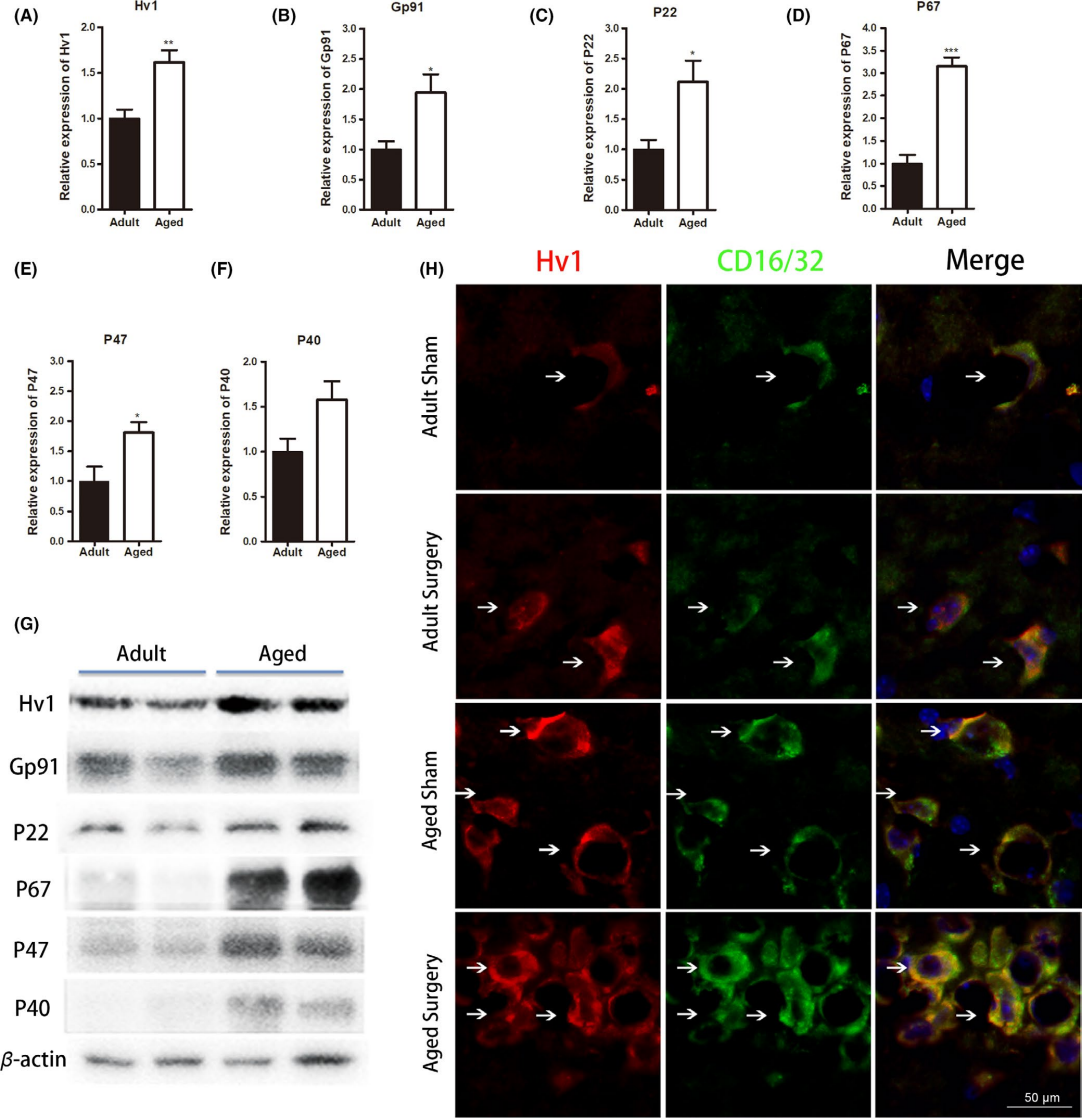
Hv1 and NADPH component activation is altered with age. A‐G, Western blot analysis revealed significant increases in Hv1 and NADPH oxidase subunits Gp91phox, P22phox, P67phox, and P47phox protein expression in aged sham compared to adult mice. H, Representative immunofluorescent images of the hippocampal dentate gyrus showing that Hv1‐positive cells co‐expressed with CD16/32 were increased in aged brain after surgery (scale bar, 50 μm). Brain sections were stained with Hv1 (red) and CD16/32 (green). All data are presented as the mean ± SEM. n = 4‐5; *P < .05, **P < .01, ***P < .001 vs adult sham, Student's *t* test

The voltage‐gated proton channel Hv1 is pivotal for high‐level NADPH oxidase‐dependent superoxide production.^17^ The recent study suggested Hv1 is one of the important factors that contribute to brain ischemic damage.^17^ Thus, we examined whether the expression of Hv1 and NADPH oxidases is associated with enhanced proinflammatory responses and synaptic dysfunction after tibial fracture surgery. We found that the expression of Hv1 was increased to 1.5‐fold in aged brain compared with adult (n = 4‐5; *P* < .01 vs adult sham; Figure 6A). Moreover, the expression of NADPH oxidases subunit Gp91phox (n = 4‐5; *P* < .05 vs adult sham; Figure 6B), P22phox (n = 4‐5; *P* < .05 vs adult sham; Figure 6C), P67phox (n = 4‐5; *P* < .001 vs adult sham; Figure 6D), and P47phox (n = 4‐5; *P* < .05 vs adult sham; Figure 6E) was significantly increased by 2‐, 2‐, 3‐, and 1.7‐fold in the aged brain as compared to adult. Tibial fracture surgery did not cause further elevation of Hv1 and NADPH oxidases subunit expression in both adult and aged animals (data not shown). However, double immunofluorescent staining revealed that the number of Hv1‐positive cells co‐expressed with CD16/32 was increased in the aged brain after surgery (Figure 6H).

## 4 DISCUSSION

Microglia‐mediated neuroinflammation plays a pivotal role in the pathogenesis of POD and POCD.^22^ Open tibial fracture surgery in 2‐ to 3‐month‐old adult mice causes hippocampal‐dependent memory impairment that is associated with increased IL‐1β expression in the hippocampus.^20^ Functional inhibition of IL‐1β, both in mice pretreated with IL‐1 receptor antagonist and in IL‐1R knockout mice, mitigated the neuroinflammatory effects of surgery and memory dysfunction.^20^ Further study showed that TNF‐α acts upstream of IL‐1β and provokes its production in the brain.^23^ In the current study, we found that tibial fracture surgery increased TNF‐α and IL‐1β levels exclusively in the hippocampus of aged mice, but not adult mice. The level of synaptophysin (SYP), a synaptic protein correlated with cognitive function, was also markedly reduced at 72 hours after surgery. These data are consistent with the findings from a previous study that peripheral surgery leads to an exaggerated neuroinflammatory response in aged mice.^6^, ^7^ These data also support the clinical observation that senior patients are more vulnerable to develop POD and POCD.^3^, ^24^

Several studies indicate that exaggerated cytokine production in the aged brain following central or peripheral stimulation is dependent on activation of microglia. For example, microglia isolated from aged mice 4 hours after LPS injection had increased mRNA levels of IL‐1β, TLR2, and indoleamine 2,3‐dioxygenase compared to adult mice injected with LPS.^25^ Similarly, microglia from aged mice stimulated ex vivo with LPS and Pam3CSK4, a TLR2 agonist, produced higher levels of IL‐6 and TNFα than microglia from young mice.^26^ Microglia in the “resting” state usually display a ramified morphology with small cell bodies and many long, thin processes. When activated, they become de‐ramified with enlarged cell bodies and retracted, condensed processes.^27^ By using the Iba1, a protein expressed on the surface of microglia, we found that microglia of aged mice exhibited thickened and de‐ramified processes compared with young adults. In agreement with the inflammatory profile, Iba1 protein levels were significantly elevated in aged hippocampus after tibial fracture surgery. These data suggest a potential role for microglia in aging‐related hypersensitivity to the “second hit” of acute peripheral surgery.

Microglia adopt different activation status to promote CNS repair and regeneration.^9^ The balance of M1/M2 phenotypes may be a determinant for inflammatory progression. In the current study, we identify age as an influential factor for regulating this balance after peripheral surgery. Both adult and aged mice exhibited significant increases in M1 activation after peripheral surgery. However, aged mice exhibited a reduced M2 polarization and increased M1 polarization compared to adults. Similar microglia/macrophage‐mediated imbalances have been reported for other age‐related neurological conditions. For example, after cerebral ischemic injury, the aged brain demonstrated M1 polarization soon after distal middle cerebral artery occlusion (dMCAO) and a long‐lasting impairment in M2 responses.^16^ There was a strong positive correlation between favorable neurological outcomes after dMCAO and myelin basic protein (MBP) levels or the number of M2 microglia/macrophages.^16^ Aged brain is also more vulnerable to the damaging effects of traumatic brain injury and that highly activated microglia/macrophage that have an M1 proinflammatory phenotype contribute to exacerbated neurodegeneration in the aged traumatic brain injury (TBI) brain.^28^ Studies from experimental spinal cord injury showed that aging primes macrophage activation toward a proinflammatory M1‐dominant phenotype with decreased M2b macrophage activation, contributing to impaired functional recovery and enhanced tissue damage in 14‐month‐old mice.^29^

The mechanism that aged animals have a reduced capacity to resolve amplified microglial activation after an immune challenge is largely unknown. One working hypothesis is that worsened injury outcome in aged mice was associated with an impaired microglial response to the M2 and repair promoting effects of IL‐4.^30^ Loss of IL‐4 decreased expression of M2 markers and then exacerbated sensorimotor deficits and impaired cognitive functions after cerebral ischemia.^31^ When activated microglia from adult and aged mice were isolated and treated ex vivo with IL‐4, only microglia from adult mice were successfully re‐directed toward an anti‐inflammatory and M2 profile with increased Arg1 and reduced inducible nitric oxide synthase (iNOS) mRNA expression.^30^ It is also suggested that the level of microglial NADPH oxidase activity is a major regulator of the shift between M1/proinflammatory and M2/immunoregulatory microglial phenotypes.^32^ Zhang et al reported that aging significantly increased NADPH oxidase activation and reactive oxygen species production in Arg1‐positive M2 microglia/macrophages, thereby further increasing age‐related, macrophage‐mediated spinal cord injury secondary tissue damage.^15^ Another study by Kumar et al demonstrated that aged mice had increased microglia/macrophage expression of major histocompatibility complex II and NADPH oxidase, and reduced antioxidant enzyme expression which was associated with worse outcome in traumatic brain injury.^28^

In the current study, we found that aged mice have upregulated Hv1 and NADPH oxidase subunit expression compared with adult mice. In the brain, the Hv1 channel is selectively expressed in microglia and is required for NADPH‐dependent ROS generation both in vitro and in vivo.^17^, ^18^ Tibial fracture surgery did not cause further elevation of Hv1 and NADPH oxidase subunit expression in either adult or aged brain, indicating that brain Hv1/NADPH is much more affected by the pathophysiological changes with aging rather than the intervention of peripheral surgery in our experimental model. The recent study suggested that Hv1 is one of the important factors that contribute to age‐dependent exacerbation of brain ischemic damage. Aged Hv1 knockout mice showed smaller neuronal damage in brain ischemia, while young Hv1 knockout animals did not show any difference compared with young wild‐type mice.^33^ A novel observation in our study is that the percentage of CD16/32‐positive M1 microglia colabeling with Hv1 was higher in aged mice compared with adult mice after tibial fracture surgery. This finding supports the concept that Hv1 might be a potential target for modulating microglial M1/M2 polarization in the aged brain. Hv1 deficiency was found to attenuate brain damage via skewing the balance of the microglial response toward a more protective phenotype after ischemia.^34^ Future studies will determine how Hv1 modulates microglia polarization in the aged brain, and what mechanisms are involved in this process.

This study has some limitations that have to be pointed out. First, the current study is comparing tibial fracture vs sham‐operated animals which were anesthetized with isoflurane. Prior studies have described altered responses to aging with isoflurane alone in nonsurgical animals.^35^, ^36^ Thus, it is possible that enhanced neuroinflammation in the aged brain after tibial fracture surgery may be due to some interaction between surgery and anesthesia, or other perioperative variables. However, it should be noted that isoflurane exposure time was relatively short in our tibial fracture surgery model (about 15‐20 minutes) compared to those reports investigating isoflurane alone. Indeed, studies from both adult and aged animals have demonstrated that surgical trauma, rather than isoflurane anesthesia, resulted in neuroinflammation, glial cell activation, and cognitive function impairment.^7^ Second, while CD16/32 and CD206 have been used as microglial M1/M2 polarization markers in this as well as many other studies,^13^, ^31^ microglia, however, can have a wide range of phenotypes with associated properties depending on their microenvironment. Further study using a combination of markers to analyze the temporal dynamics of microglia polarization will advance our understanding of brain microglia states following peripheral surgery. Third, we demonstrated that the percentage of CD16/32‐positive M1 microglia colabeling with Hv1 was higher in aged mice. However, the specific pharmacological Hv1 channel inhibitor is currently unavailable. The direct cause‐and‐effect relationship between elevation of Hv1/NADPH and imbalance of microglia M1/M2 polarization in the aged brain following peripheral surgery could not be concluded in the current experiment. Future investigations using genetically modified animals will help to elucidate the mechanisms underlying our findings.

In conclusion, the present study provides novel evidence that greater neuroinflammation and deterioration in synaptic function from aged mice after peripheral surgical intervention was associated with Hv1/NADPH oxidase upregulation, which may shift the dynamic equilibrium of microglial activation toward a proinflammatory M1 polarization state.

## CONFLICT OF INTEREST

The authors declare no conflict of interest.

## ETHICS APPROVAL AND CONSENT TO PARTICIPATE

The Ethics Committee at Peking University Shenzhen Hospital Animal Care and Use Committee (Shenzhen, China) approved the protocols of this study. All experiments were conducted in accordance with the National Institutes of Health Guide for the Care and Use of Laboratory Animals.

## CONSENT FOR PUBLICATION

Yes.

## Data Availability

The datasets used and/or analyzed during the current study are available from the corresponding author on reasonable request.
